# Diversity of Tanaidacea (Crustacea: Peracarida) in the World's Oceans – How Far Have We Come?

**DOI:** 10.1371/journal.pone.0033068

**Published:** 2012-04-04

**Authors:** Magdalena Blazewicz-Paszkowycz, Roger Bamber, Gary Anderson

**Affiliations:** 1 Department of Polar Biology and Oceanobiology, University of Łódź, Łódź, Poland; 2 Artoo Marine Biology Consultants, Ocean Quay Marina, Southampton, Hants, United Kingdom; 3 Department of Biological Sciences, University of Southern Mississippi, Hattiesburg, Mississippi, United States of America; University of Guelph, Canada

## Abstract

Tanaidaceans are small peracarid crustaceans which occur in all marine habitats, over the full range of depths, and rarely into fresh waters. Yet they have no obligate dispersive phase in their life-cycle. Populations are thus inevitably isolated, and allopatric speciation and high regional diversity are inevitable; cosmopolitan distributions are considered to be unlikely or non-existent. Options for passive dispersion are discussed. Tanaidaceans appear to have first evolved in shallow waters, the region of greatest diversification of the Apseudomorpha and some tanaidomorph families, while in deeper waters the apseudomorphs have subsequently evolved two or three distinct phyletic lines. The Neotanaidomorpha has evolved separately and diversified globally in deep waters, and the Tanaidomorpha has undergone the greatest evolution, diversification and adaptation, to the point where some of the deep-water taxa are recolonizing shallow waters. Analysis of their geographic distribution shows some level of regional isolation, but suffers from inclusion of polyphyletic taxa and a general lack of data, particularly for deep waters. It is concluded that the diversity of the tanaidomorphs in deeper waters and in certain ocean regions remains to be discovered; that the smaller taxa are largely understudied; and that numerous cryptic species remain to be distinguished. Thus the number of species currently recognized is likely to be an order of magnitude too low, and globally the Tanaidacea potentially rival the Amphipoda and Isopoda in diversity.

## Introduction

Tanaidacea is an order of crustaceans of the superorder Peracarida, which includes species, that are among the smallest of the benthic macroinvertebrates. Their body lengths rarely exceed 2 mm, although the largest representative, *Gigantapseudes maximus* (for all authorities see WoRMS [1]) reaches over 7 cm in length. Tanaidaceans are truly demersal organisms which mainly inhabit the surface layer of the sediments, either in burrows, or by constructing tubes, or interstitially e.g. [2], [3]; some of the taxa are crevice dwellers, and others build tubes on algae or even on marine vertebrates (*Hexapleomera robusta*); one species (*Charbeitanais spongicola*) lives within sponge ostia, and at least one species (*Exspina typica*) is parasitic in holothurians [4].

Tanaidacea may be considered an eurytopic group, recorded from almost any type of marine habitat: from hyperhaline lakes to hypohaline interstitial zones, saltmarshes and mangroves [5], [6], [7] and to the deepest abyssal habitats. They have been recorded in underwater caves [8], and at chemosynthetic habitats such as hydrothermal vents [9] mud volcanoes [10], [11] and sea-bed pock-marks [12]. Additionally, four species to date have been recorded from freshwater habitats [13].

**Figure 1:**
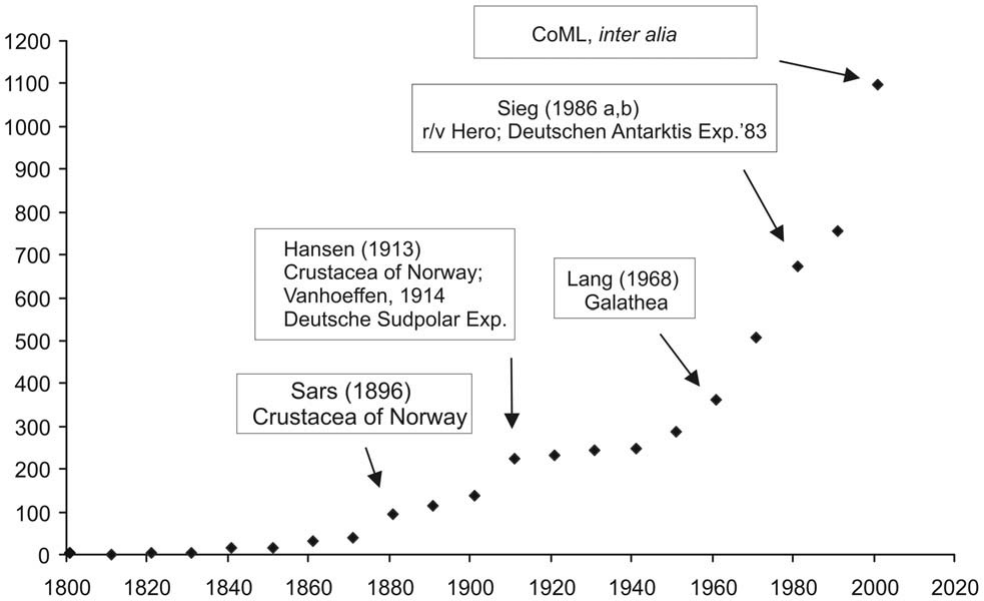
Cumulative number of tanaidacean species described in each decade since 1800.

The history of the Tanaidacea goes back to over 200 years ago to the first species discovered. These specimens appear to be of a member of the present-day family Leptocheliidae, but since Tanaidacea were unknown to science at that time the species was identified as an amphipod – *Gammarus heteroclitus* Viviani, 1805. The type material no longer exists, therefore it is accepted that the first valid tanaidacean species was *Apseudes talpa* described by Montagu (as *Cancer Gammarus Talpa* [14]). Tanaidacea had an unclear status until the 19th century, commonly being classified within the Isopoda (tribe Chelifera) or occasionally within the Amphipoda. They were given separate ordinal status by Hansen [15]. Currently the order includes almost 1200 described species. The cumulative number of tanaidacean species described in the past decades is presented in Figure 1; we suggest that the present number of nominal species is a gross underestimate of the actual number of species.

In terms of higher phylogeny, there is lack of consistency in opinion of the putative sister group to the tanaidaceans. Analyses based on morphology, anatomy and postmarsupial development have shown affinities with isopods or cumaceans [16], [17] they have appeared as a distinct branch in cladograms, but also as a paraphyletic group with the cumaceans. The comprehensive analysis of Poore [18] consistently found the Tanaidacea to be a sister-group to the Cumacea; some recent molecular approaches have questioned the Tanaidacea as a monophyletic group [19], [20]; the distinct suborders (see below) are consistently thought to be monophyletic, but a recent analysis of [21] suggested that the Neotanaidomorpha may lie within the Tanaidomorpha.

**Figure 2:**
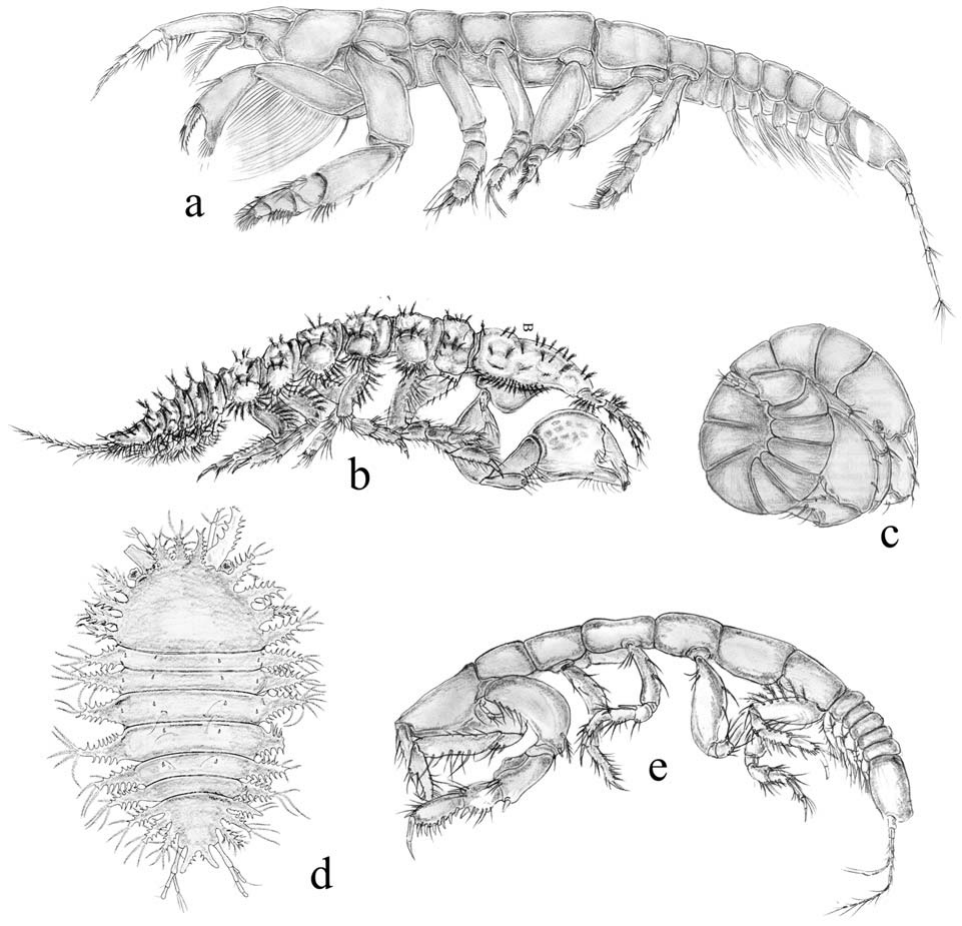
Apseudomorphan adaptations. a, *Kalliapseudes* sp. with a filter-feeding apparatus on the chelipeds; b, *Cyclopoapseudes* sp., with a flattened body adapted to burying in the upper layer of soft sediments; c, *Macrolabrum* sp., with its twisted posterior thorax and abdomen, adapted to living inside empty snail shells, d; *Tanzanapseudes* sp., with a flattened body adapted to life on and between flat surfaces of coral rubble; e, *Bunakenia* sp with its pereopods adapted to digging in soft sediments.

**Figure 3:**
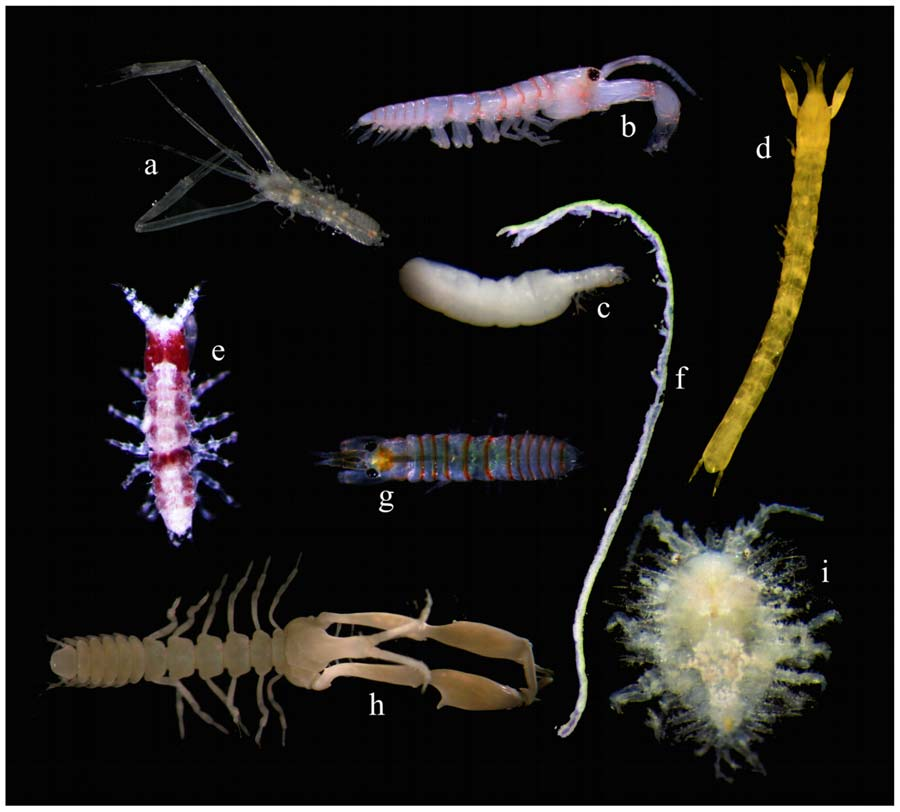
Diversity of forms in the Tanaidacea. a–b, g, various forms of males in the Leptocheliidae; c, female of *Pooreotanais* sp.; d, *Arhaphuroides* sp.; e, *Sinelobus* sp.; f, Anarthruridae indet.; h, *Neotanais* sp.; i, *Metatanais* sp.; j, *Tanzanapseudes* sp.

Conventional taxonomy divides the Tanaidacea into four suborders: Anthracocaridomorpha, Apseudomorpha, Neotanaidomorpha and Tanaidomorpha. The first includes only fossil species, and will not be discussed further here. The Apseudomorpha is considered to be the most plesiomorphic, with a fossil record from the Lower Carboniferous, the Triassic and the Jurassic [22]. This group is represented by 457 currently-recognized species in about 93 genera, and is widely represented in various shallow marine benthic habitats on silt, sand or gravel. Apseudomorphs are also abundant in coral reefs, estuaries and mangroves from the tropics to temperate waters; only a few of them (e.g. the subfamily Leviapseudinae) are almost exclusively deep-sea taxa. Apseudomorphs are morphologically highly diversified in adaptation to a range of particular and specialized habitats, from the free-living forms, to genera adapted to filter-feeding, subfamilies adapted to an interstitial mode of life or to living in empty gastropod shells, and families such as the Tanzanapseudidae which are adapted to living on flat surface of coral rubble (Figure 2, Figure 3i, j).

The Neotanaidomorpha is a less diverse suborder with 45 species classified into four genera and a single family. The representatives are exclusively deep-sea forms, and, except for the Antarctic region, they have never been recorded at shelf depths. The mature males in this suborder demonstrate sexual dimorphism that is characterized by their highly-modified chelipeds (Figure 3h); the function of these oversized chelipeds is unknown.

The fourth suborder, the Tanaidomorpha, represented by about 550 described species, 120 genera and about 18 families (currently under major revision), shows the least gross morphological diversity (Figure 3a–g). Their bodies are consistently elongate, usually oval in cross section, and covered by smooth cuticle. Their external morphology is simplified in comparison with those of the other two suborders, which is consistent with their predominantly tubicolous life-style. It is assumed that these most-apomorphic tanaidomorphs are able to produce tubes from excretions of tegumental glands. The tubes are occasionally encrusted by sedimentary particles [23] and are the structures within which the animals conceal themselves and their broods. Females are less motile than the males. They are presumed to stay inside the tube for their whole lives including during reproduction. The first free-living juveniles, the mancae, leave the marsupium but stay inside the maternal tube for a few days, and then leave them through pores drilled in the walls [24]. Because mancae have a limited swimming ability, they usually settle and construct tubes in close proximity to the maternal tube.

## Methods

Data and information collated and analyzed herein were gleaned from the gamut of published literature and databases, and particularly from WoRMS [1].

Global distributional analysis at the species level was considered pointless, because the distributions of few if any species encompass more than one ocean basin. Global distribution was therefore analyzed based on the number of species per higher taxon (normally genus) present in each zoogeographic region, by nearest-neighbour cluster analysis, derived from the % Bray-Curtis similarity of the regions, using the CAP package of Pisces Conservation Ltd. This approach is considered more informastive than simple presence-absence analysis by genus, as it also takes into account the diversity of a genus within an area; thus, the assumption is made that two areas each of which has ten species of *Apseudes*, say, will be more similar to each other than either will be to an area with only one species of *Apseudes*.

Owing to the low number of species per genus in the Tanainae, the Sinelobinae, the Sphyrapinae and the Neotanaidomorpha, these taxa have been used in the analysis at the subfamily (the first three) or suborder (the last) level, in order to maximize the dataset. Similarly, the two subgenera of the relatively speciose genus *Zeuxo* (*Zeuxo* and *Parazeuxo*) have been included separately, as have the two groups of *Leptochelia*, the “*savignyi*-group” and the “*minuta*-group”.

Shallow waters (<200 m depth) and deep waters have been treated separately.

## Results and Discussion

### Dispersion and distribution

While α diversity is a measure of occupied available niches within a habitat or region, its development depends on initial colonization. Tanaidaceans, as other peracarids, brood their young, which are released as crawling mancae, effectively a miniature version of the adult. They, as the adults, have no obligate dispersive ability. Only in males of some paratanaoidean families is there any significant swimming ability e.g. [25]. As a result, tanaidaceans have a minimal inherent dispersive ability. While this has not been specifically studied, research on the ecologically and morphologically similar (but generally larger) tubicolous anthurid isopod *Cyathura polita* found individuals to travel an average of 2 m in total during their lifetime [26]. As a result, any significant dispersion in these taxa must be passive. Equally, populations are inevitably isolated and will undergo allopatric speciation at least by random genetic drift, leading to high regional diversity.

It is therefore apparent that the idea of cosmopolitan tanaidacean species suggested in the 1970s and 1980s e.g. [27], [28] are quite untenable without some significant non-tanaidacean dispersion vector. Four potential causes of passive dispersion are feasible for tanaidaceans.

#### Anthropogenic transport

The first of these is not considered to have had sufficient time to lead to allopatric speciation, but may result in apparently widespread distributions of some species. The presence of the Eritraean kalliapseudid *Cristapseudes omercooperi* in the eastern Mediterranean is most likely to be associated with transport via shipping through the Suez Canal e.g. [29]. Equally, the presence of at least three species in the Macaronesian archipelagos was attributed [30] to possible transport on ships' hulls, including that of the north-east Atlantic/Mediterranean species *Tanais dulongii*, which has also been carried as far afield as Western Australia [28]; Edgar pers. comm.). Successful introduction by this means depends on the transported population arriving in a suitable habitat: it would not be a process by which tropical species could be introduced to temperate waters, for example, as they would not survive.

#### Transport on marine vertebrates

One species of the Tanaidae, *Hexapleomera robusta*, has been found living in relatively high densities in its tubes on the carapaces of marine turtles, and also on manatees, as well as possibly in the benthos [31], [32], [29]. This behaviour has the potential for a wide distribution, or at least as wide as that of the individual vertebrate, and as a result this species was assumed to show a cosmopolitan distribution e.g. [28]. While marine turtles do not show a cosmopolitan distribution as populations, let alone as individuals, this process has potential for wide distribution of commensal tanaidaceans until they become detached from their host. Nevertheless, more recent detailed taxonomy (Bamber, unpubl.) has discovered that at least four species of *Hexapleomera* exist globally, and probably more given critical examination of previously synonymized collections. No other tanaidacean taxa are known to show such a commensalism.

#### Transport in floating algae, or similar (rafting)

Certain tanaidaceans can feasibly complete a successful life-cycle amongst floating weed such as *Sargassum*, and indeed the presence of the tanaid *Zeuxo exsargasso* in the Canary Islands is attributed to this process [33] Bamber [34] modelled the potential of this process for dispersion around the Indo-West Pacific, including an indication of high diversity resulting in such areas as southern Australia (now known to show high tanaidacean diversity), the South China Sea and Japan. Dispersion by this means will depend on prevailing sea-currents, and will not be available to most infaunal or any deep-water species.

#### Geological transport

Geological transport involves plate-tectonics and sufficient time for population isolation and allopatric speciation in non-mobile marine taxa; this is the only one of these four processes to operate in the deep sea. This process has been suggested for the dispersion of, and speciation within, genera endemic to chemosynthetic seabed-habitats [10]. The aberrant genus *Coalecerotanais* is a specialized inhabitant of chemically-reduced habitats, being recorded from cold-seeps; the known distribution of its three species is the Gulf of Mexico [2], the Gulf of Cadiz [10] and the Angola Slope (Bamber, unpubl.). Błażewicz-Paszkowycz *et al.* [10] postulated the evolution of the genus on the proto-Mid-Atlantic Ridge in the early Mesozoic era, before the separation of the Atlantic; subsequent separation of the European and North American Plates isolated populations at appropriate mud-volcano- or seep-habitats (only), resulting in isolated widely-distributed populations and allopatric speciation.

Of these four processes, the first two are likely to contribute to wider distributions of species, while the second two are likely to introduce taxa to new habitats (including creating new habitats) but also to isolate populations allowing them to diversify and speciate. Without these processes, slow spread followed by population isolation and allopatric speciation are expected to be inevitable in the Tanaidacea, resulting in high global diversity. Other than possibly *Hexapleomera* spp., the suggestion that tanaid species such as *Sinelobus stanfordi* are cosmopolitan should be subject to close and competent re-examination. Such “species” are likely to be aggregates of sibling species.

### Diversity with depth

While tanaidaceans occur from the littoral to the abyss, genera are rarely, and species never, eurybathic. As a rule, species from deep waters are blind, usually without eyelobes; as it is presumed that the presence of eyes is plesiomorphic, this character is considered of value in understanding the evolutionary depth-distribution history of taxa.

#### Suborder Apseudomorpha

**Figure 4:**
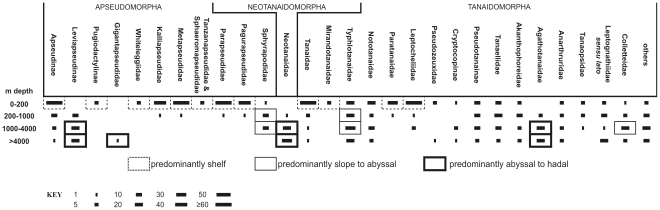
Numbers of species by four depth-bands for most families of the Tanaidacea (some monotypic families excluded for clarity), arranged by suborder. The four depth bands are essentially littoral to shelf, slope, lower bathyal to upper abyssal, and lower abyssal to hadal.

The apseudomorphs are predominantly shallow-water taxa, with most only extending their distribution to slope depths. Eight of the families (Figure 4) seldom exhibit a bathymetric range exceeding 200 m. Most of these taxa have functional eyes.

The Apseudidae is regarded as the most plesiomorphic family, and has therefore had more time to disperse (bathyally), evolve and adapt. Within this family, the subfamily Apseudinae remains predominantly shallow-water, 60 of its 82 species being recorded from shallower than 200 m, while only three species are found below 4000 m. Conversely, the Leviapseudinae is a deeper-water subfamily of blind species, never occurring above 200 m, and only eight of its 48 species recorded shallower than 1000 m; this subfamily is assumed to have diverged from the Apseudinae in deep water and diversified.

One deep-water family is the Gigantapseudidae, consisting of two large, abyssal species in one genus. They are morphologically similar to Leviapseudinae, from which they probably evolved. Another is the more diverse Sphyrapodidae. Other than two congeneric shallow-water species, sphyrapodids are predominantly lower bathyal to abyssal in distribution, although they may approach shelf waters in northern (but not southern) polar waters. This family exhibits a distinctive morphology, although remotely similar to the distinctly shallow-water kalliapseudids. The Sphyrapodidae is presumed to have diverged from some protoapseudid stock long ago and evolved in the deep sea.

Thus, overall, the Apseudomorpha is highly diverse in shallow waters, including the littoral zone, and evolutionarily has diversified into deep waters at least twice.

#### Suborder Neotanaidomorpha

**Figure 5:**
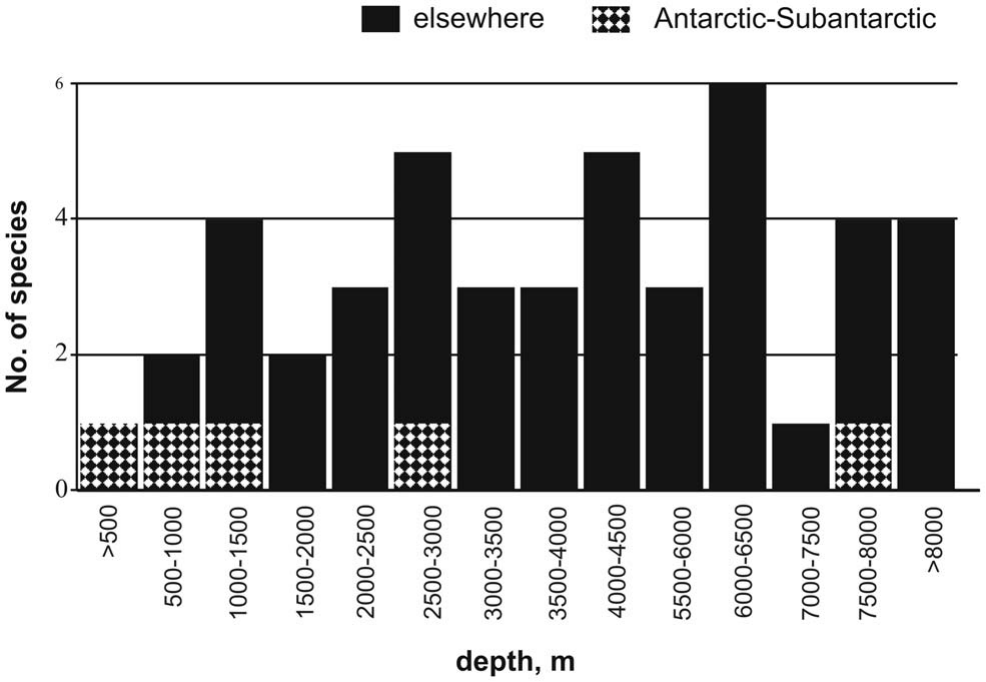
Number of species of Neotanaidomorpha in various depth bands.

As stated above, the Neotanaidomorpha is a distinctly deep-water suborder. Depth ranges are difficult to define precisely, owing on the one hand to the scarcity of most of the species (often known from only one sample-location) and on the other hand to the uncertain and wide depth ranges of deep-water bottom-trawl samples. Neotanaidomorphs are found across the full range of the deep-sea floor depths, down to over 9000 m, with only a few recorded from shallower than 1500 m, these latter being mostly Southern Ocean species (Figure 5). When one appreciates the historic size of sampling effort at different depths (basically an inverse relationship), it is apparent that this suborder is predominantly abyssal to hadal in distribution. While most (38) of the 48 species are in the genus *Neotanais*, the remaining genera show no particular depth distinction; rather, the entire suborder is diverse in the deep sea.

#### Suborder Tanaidomorpha

The depth distribution of the Tanaidomorpha is more complex (Figure 4). Four families are predominantly shallow-water, their few deeper-water representatives (if any) presumed to have moved down secondarily.

The superfamily Tanaoidea includes one family, the Tanaidae, comprising some 68 species in 19 genera, mostly of a consistent gross morphology. The subfamily Langitanainae includes six species of very modified morphology, four of these extending into slope depths. Most occur in polar waters (e.g. *Langitanais* spp.), the exception being the aberrant carnivore *Mekon solidomala*, which occurs in upper-slope waters off New Caledonia. Within the subfamily Tanainae, one of the two species of *Synaptotanais* is known only from a depth of 1300 m off Indonesia, and the two species of the genus *Protanais* are recorded from abyssal to hadal depths. All of these species have eyelobes, although four are without eye-pigment, reinforcing the belief that they have evolved from shallow-water ancestors. All of the remaining 61 species of Tanaidae (15 genera), are from littoral and shallow-shelf waters. They have functional eyes and usually have dorsal pigmentation in complex patterns. The commonest littoral taxon, *Zeuxo*, exhibits allopatric speciation: so far, three distinct species are known from identical habitats on the North Atlantic coasts of Europe (from SW England to Portugal) and in Macaronesia. None are sympatric. Ten species have been described from the southern and eastern temperate coasts of Australia [35], [63]. This is evidently a diverse shallow-water family with some recent secondary colonization of deeper waters.

Within the superfamily Paratanaoidea, two families, the Paratanaidae and the Leptocheliidae, show a similar pattern to the Tanaidae, with high diversity of eyed species in littoral to infralittoral habitats, and occasional deeper-water secondary colonizers. The Leptocheliidae, once thought to include a few cosmopolitan species e.g. [37], has more recently been found to comprise numerous species showing a high degree of allopatric speciation and niche specificity [36]. Most of the 62 species are littoral, many extend to about 50 m depth and a few to 100 m. The exceptions are in two genera. The monotypic genus *Bathyleptochelia*, which includes *Bathyleptochelia oculata*, has functional eyes and was collected from 570 m depth in the Gulf of Mexico. Six of the seven described species of *Mesotanais* are found below 800 m (one to 1700 m); all are without functional eyes, but do have eyelobes (see [10] fig. 23C, D), again supporting the hypothesis that this is a very diverse shallow-water family with a few taxa showing recent secondary colonization of deeper-waters.

The Paratanaidae, with 28 recorded species, includes by contrast few littoral species, although of those the two species of *Teleotanais* show a dorsal pigmentation similar to that found in the Tanaidae. Most species occur from the shallow-sublittoral to about 50 m depth; only three species are recorded from greater than 200 m depth (260 to 591 m); all paratanaid species have functional eyes. This is again a shallow-water family with little (but recent) colonization of deeper waters.

The third predominantly shallow-water paratanaoid family is the Mirandotanaidae, which comprises three blind species of aberrant morphology. Their life-style is quite unknown, but parasitism has been suggested (and disputed – see [36]). All occur in shallow water, although one extends to 580 m depth.

Two additional families have an apparent shallow-water bias to their distribution. The Tanaopsidae (new family diagnosis [39]), incorporating species of the genus *Tanaopsis*, are all blind, and the genus extends in distribution from the intertidal to over 3000 m, although most species occur above 1000 m. Their affinities are unclear, but they may have evolved from a leptognathiid *sensu lato* ancestor on the lower shelf and subsequently extended to both shallower and deeper waters. The Nototanaidae as currently understood is believed to be polyphyletic; the type genus, *Nototanais*, comprises eyed species from Antarctic shelf depths; other genera are shallow to littoral, eyed or blind, and some with more affinities to the Typhlotanaidae are from slope to bathyal waters and blind.

The remaining families largely extend across most of the depth range (shallow to deep), and most are blind. They include many deep sea forms; indeed, Larsen [2] stated that “tanaidaceans have their greatest ecological importance on the abyssal plain where they might even rival that of the polychaetes”. In slope waters, a recent survey off Brazil at around 700 m depth found three species of tanaidacean to be among the nine dominant species, compared with two polychaete species and four amphipod species, out of a total of over 400 taxa *vide* [40].

Within the family Pseudotanaidae, there are a number which are exclusively eyed shallow-water genera, and more which are exclusively blind deep-water genera. The largest genus, *Pseudotanais*, with perhaps 37 species, is in itself interesting, in that it includes nine eyed species, ranging in depths from 0 to 121 m, and a majority of blind species, found from 20 to over 7000 m, but mainly below 250 m. This again appears to be a group which has evolved in shallow water, but which has achieved its greatest diversity in deep waters.

The remaining families are largely found from shelf depths to deep water, with their greatest diversity in slope to abyssal depths. It is accepted that their diversity in the deep-sea is currently severely underestimated. Such families as the Typhlotanaidae, the Tanaellidae, the Anarthruridae and the Colletteidae are believed to have evolved in deep water and some have secondarily moved to shallower waters. The exception is the Agathotanaidae, which only comprises deep-water species, with 41 of its 48 species occurring deeper than 1000 m.

We may therefore postulate that tanaidaceans first evolved in shallow waters, the region of greatest diversification of the Apseudomorpha and some tanaidomorph families, while in deeper waters the apseudomorphs have subsequently evolved two or three distinct phyletic lines. The Neotanaidomorpha has evolved separately (and diversified globally), and the Tanaidomorpha has undergone the greatest evolution, diversification and adaptation, to the point where some of the deep-water taxa are recolonizing shallow waters.

### Diversity and zoogeography

One third of the 109 genera in the Apseudomorpha (34 genera) have their distributions constrained to one, usually well-defined, ocean basin (Table 1). For example the distributions of 28 of these occur in the Indo-Pacific and/or Australian region, occasionally extending to remote Pacific locations such as Vanuatu, Hawaii or the coast of Japan. Only three genera (*Pectinapseudes, Pseudoapseudes, Vestigiramus*) have their distributions restricted to the central Atlantic.

**Table 1:** Selected families and genera of the Apseudomorpha that exhibit restricted zoogeography.

Name	number of species	distribution
MIRANDOTANAIDAE	3	Antarctic/Australia
TANZANAPSEUDIDAE	9	Indopacific
WHITELEGGIIDAE	3	Australia
NUMBAKULLIDAE	2	Indopacific, Australia
APSEUDIDAE		
*Bilobatus*	3	Indopacific, Australia
*Glabroapseudes*	4	Atlantic
*Pectinapseudes*	3	NE Atlantic, Gulf of Mexico
*Pugiodactylus*	5	Indopacific, Antarctic
*Spinosapseudes*	2	Tasman Sea
*Xanthapseudes*	2	Australia
*Colobocladus*	2	Indian Ocean
PARAPSEUDIDAE		
*Longiflagrum*	5	Australia/Indian Ocean
*Biropalostoma*	3	Indopacific, Australia
*Ctenapseudes*	3	Indopacific
*Magniaculeus*	3	S Australia
*Brachylicoa*	4	Indopacific
*Gutuapseudes*	2	Indopacific, NW Australia
*Pseudoapseudes*	2	Caribbean
METAPSEUDIDAE		
*Jumarichardia*	5	Indian Ocean
*Vestigiramus*	2	Carribean, SW Atlantic
*Metapseudes*	2	Australia
*Msangia*	3	Indopacific, Australia
*Cryptapseudes*	3	Indopacific
*Curtipleon*	4	Indopacific, Australia
PAGURAPSEUDIDAE		
*Indoapseudes*	4	Indopacific, Australia
*Hodometrica*	2	Hawaii, Australia
*Similipedia*	2	Indian Ocean, Australia
*Pagurapseudes*	11	Indian Ocean, Australia
*Macrolabrum*	9	Indian Ocean, Australia
*Pagurapseudopsis*	5	Indopacific, Australia
KALLIAPSEUDIDAE		
*Hemikalliapseudes*	3	Equatorial East Atlantic

In this context, it is worth emphasizing that 28 of the 109 genera mentioned above are monotypic and their distributional analysis is infeasible.

The seven exclusively deep-sea apseudomorph genera (*Glabroapseudes*, *Langapseudes*, *Carpoapseudes*, *Colobocladus*, *Eliomosa*, *Fageapseudes*, and *Leviapseudes*) have distributions scattered through more than one oceanic basin.

*Gigantapseudes* (fam. Gigantapseudidae) has been recorded in the Pacific, but since it is known from only two species and two records, no reasonable conclusions can be drawn about its distribution and origin. More intriguing is the distribution of the Sphyrapodidae, which, except for two North Atlantic species (*Sphyrapus anomalus* and *Sphyrapoides bicornis*), is a deep-water family (see above). Nevertheless, this is the only family so far which has never been recorded below the Antarctic convergence. As the family is considered to be phylogenetically young it is suggested that its members might have invaded the deep sea a relatively short time ago. It may have taken place in the North Atlantic, from where they were transported by the global conveyor (the oceanic thermohaline circulation) to more remote places, followed by allopatric radiation. A recent time for that event, presumably at the Eocene/Oligocene boundary, when the Antarctic Circumpolar Current (ACC) began, would explain the absence of sphyrapodids in the Antarctic.

With respect to distribution, the Tanaidomorpha is divided into two clear groups. The first comprises the Tanaidae, Pseudozeuxidae, Paratanaidae and Leptocheliidae. These are generally considered to be plesiomorphic tanaidomorphs; they show pan-tropical and pan-temperate distributions, and can be numerous and dominant in benthic assemblages. These families never occur below the Antarctic Convergence, although some (e.g. *Langitanais* species, *Pseudoleptochelia antarctica* and *Zeuxo ohlini*) are known from a few records in sub-polar regions such as the Scotia Arch, Kerguelen Island and the Heard Island.

The second group of tanaidomorphs, comprising 11 families (including the Akanthophoreidae and the Tanaopsidae) exhibit wide distributions and are almost never restricted to one basin. They are an infrequently found in benthic assemblages in shallow waters of low latitudes, but may be abundant in the zoobenthos of polar ecosystems; below shelf depths these tanaidomorphs are commonly less numerous, but their diversity is surprisingly high.

Tanaidaceans belonging to this second group are almost exclusively blind. The exceptions are members of polyphyletic family Nototanaidae, which include *Nototanais antarcticus* and *N. dimorphus*. They are the only members of their genus, and are the most common tanaidaceans below the Antarctic Convergence, and have never been recorded above it.

The genus *Peraeospinosus*, currently represented by 13 species (one in press [39]), is a cosmopolitan, mostly deep-sea genus [41] However, in the Antarctic, its members have successfully colonized the Antarctic shelf and are currently represented there by seven species. Other than in the Southern Ocean, southeastern Australian waters (the Bass Strait) is the only locality where *Peraeospinosus* has been found at shelf depths. It is feasible that this genus could have colonized the shelf in the Early Cretaceous, when Australia, together with Antarctica, was inside the Antarctic Circle. This calls into question the earlier hypothesis that *Peraeospinosus* might have colonized the Antarctic after the isolation and full glaciation of Antarctica (∼20 my BP) [41].

The only truly cosmopolitan tanaidacean is *Exspina typica*, which was recently demonstrated to be a parasite of at least six different species of holothurians [4]. However, morphological observations to date are insufficient to determine whether its remote populations are conspecific or separate species.

#### Cluster analysis of distributional data

The shallow-water analysis is based on areas which accord with the Marine Ecoregions of the World (MEoW) provinces of Spalding *et al.* (2007: Box 1) with necessary amalgamation where data are sparse, as shown in Table 2. The deep areas accord with the MeoW lower bathyal provinces of Vierros and coauthors [42], using their terminology, except that we distinguish the Caribbean and Gulf of Mexico (GoM) from the North Atlantic, we include the Mediterranean (not clearly part of the provinces of Vierros *et al.* [43]), and we distinguish the southern half of the Indian Ocean (as recommended by Vierros *et al.*, [43] p. 35). Areas with insufficient data (e.g. Indo-Pacific deep waters, south-west Atlantic shallow waters) were excluded from the analysis, leaving 16 shallow areas and 9 deep areas.

**Table 2:** Shallow-water zoogeographic areas analyzed, compared with MeoW provinces and regions [43].

Analyzed areas	Provinces [43]	Realms [43]
N Atlantic	2, 3, 5	Temperate N Atlantic
Mediterranean	4, 7	Temperate N Atlantic
Caribbean GoM	6, 12	Temperate N Atlantic/Tropical Atlantic
W Pacific	8, 9, 28, 29, 31	Temperate N Pacific/Central Indo-Pacific
SW Atlantic	14, 47	Temperate South America
NW Indian Ocean	18, 19, 21	Western Indo-Pacific
SW Indian Ocean	20, 51	Western Indo-Pacific/Temperate Southern Africa
NE Indian Ocean	23, 24	Western Indo-Pacific
S China Sea	25	Central Indo-Pacific
Indonesia-Malaya	26, 27, 30	Central Indo-Pacific
N Australia	32, 33, 34	Central Indo-Pacific
Equatorial Pacific	35, 37 to 42	Eastern Indo-Pacific
S Australia	55, 56, 57	Temperate Australasia
New Zealand	53, 54	Temperate Australasia
Subantarctic	48, 59, 60, 62	Temperate S America/Southern Ocean
Antarctic	61	Southern Ocean

**Figure 6:**
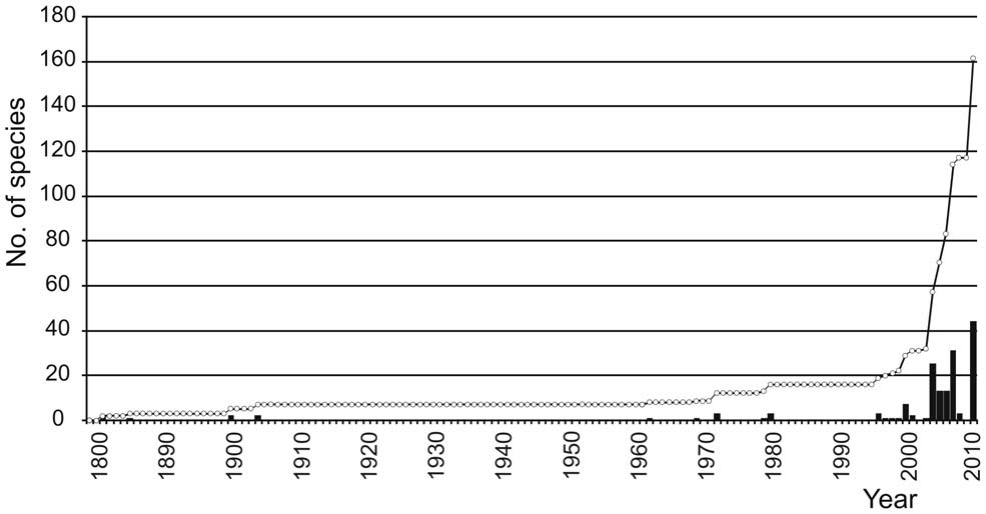
Cluster analysis of Bray-Curtis similarities (%) between various sea-areas, based on the numbers of species of tanaidacean genera (or higher taxa, see text) per region, for: a, shallow waters, <200 m depth, and: b, deep waters. A, B and C are clusters referred to in the text.

Cluster analyses of the results are shown as Figure 6. The shallow-water clustering (Figure 6a) shows poor levels of similarity, predominantly below 40%. There is some association of the Caribbean (including the Gulf of Mexico), the North Atlantic and the Mediterranean (cluster A), perhaps relating to the North Atlantic subtropical gyre. There is also a weak association between the nine Indo-West Pacific and Australasian areas (cluster B). Finally there is a slight clustering of the Subantarctic and New Zealand (and the SW Atlantic) (cluster C), perhaps relating to the subantarctic West Wind Drift. Notably, the Antarctic is completely isolated. The deep-water clustering (Figure 6b) shows higher similarity levels, with illogical associations, but the similarities are almost entirely due to four taxa, the widespread Neotanaidomorpha and *Leviapseudes*, both apparently validly pan-oceanic, and the genera *Leptognathia sensu lato* [44] and *Typhlotanais sensu lato* [3], both certainly polyphyletic. These results largely appear to demonstrate some level of regional isolation, but suffer from inclusion of polyphyletic taxa and a general lack of data, particularly for deep waters.

### The state of knowledge

In reality, it is apparent that our current knowledge of the number of tanaidacean taxa in the world, as well as their inter-suborder phylogeny, is significantly underdeveloped. From the above, we now know:

- that there is undoubtedly a great diversity of the tanaidomorphs in deeper waters yet to be discovered. It is apparent that we already know of large numbers of tanaidomorph species in the deep sea, of restricted distribution. Yet this information has come from a limited amount of sampling, and inadequate competent taxonomy of those samples. Recent studies on comprehensive surveys of deeper waters have found 36% of 59 species in the Gulf of Mexico to be undescribed [2]. Much smaller fractions of the described species are recorded on the Antarctic slope or abyssal (Amundsen Sea, Scotia, Ross and Weddell Sea) where they usually make up no more than 15% (Błażewicz-Paszkowycz, unpubl.). In areas with some greater history of study, at least 26% of tanaidomorph species from the Gulf of Cadiz were found to be undescribed [11], four of 11 species from the Norwegian Margin were discovered to be new [12], and seven new tanaidomorph species were described out of 19 species from the North-east Atlantic margin [45], [46], [47], [48], the remaining known taxa being local or endemic to these regions. Studies in progress on the equatorial West African slope (Bamber, unpubl.) are distinguishing 95% of some 88 species as new, the others being endemic to the same region, while all of a large number of tanaidaceans from the West Australian slope (Poore *et al.*, unpubl., see below) are as yet undescribed species. Geographically, almost no species are yet known from deep waters in the Mediterranean Sea, the Indian Ocean, or the South Pacific Basins. We conclude that the large majority of deep-sea tanaidaceans are yet to be discovered and described.
- that tanaidaceans are small. The historic use of coarser meshes in gear or in sample treatment will have missed many (most?) tanaidaceans. The use of 500 µm-mesh sieves for analysis of soft-sediment benthos is entirely appropriate at the community level, yet will inevitably lose a significant proportion of the tanaidacean fauna, particularly interstitial species. Use of a 250 µm-mesh in sandy substrata off Israel [29] discovered five new species, including a species of *Tanaissus*, a genus previously unrecorded from the Mediterranean, yet represented by over 7000 individuals! Poore *et al.* (unpublished), using a 0.3 µm-mesh sieve to process a collection taken in the deep sublittoral and upper continental slope (100–1000 m depth) off West Australia discovered 292 species of Tanaidacea which were dominant in number over both the Amphipoda and Isopoda. Recent sampling of the interstitial sublittoral fauna off the Azores, using a 250 µm-mesh sieve (Bamber, unpubl.), has found at least five new species, doubling the number of species from waters <200 m depth in this archipelago. Fortunately, competent deep-sea studies are biased towards using finer meshes. For example, [48] a comparative analysis of two box-core samples, found that a 250 µm-mesh retained 371 individuals and 82 species of deep-sea peracarids, compared with 164 individuals and 61 species in a 500 µm-mesh (see also [49], [50]). Unfortunately, shallow-water studies seem to be biasing in the opposite direction e.g. [51] owing to misguided economic priorities.
- that there are a great many cryptic or sibling species in shallow and deeper waters yet to be discovered or distinguished;

In a number of genera, species identification is still based on the gross morphological characters used by early workers in the 19th and early 20th centuries, while details of mouthpart and pereopod morphology and spination, known to distinguish species in other genera, have yet to be characterized. At the same time, a number of taxa still suffer from a misconception of intraspecific variability promulgated by such workers as Lang and Sieg (amongst others), who did not consider the effects of zoogeographic isolation on regional speciation. The use of molecular taxonomy for species identification is still in its infancy for the Tanaidacea [52]. Within other peracarid orders, recent work in this field has resulted in distinguishing cryptic species in the Amphipoda, using CO1 to indicate numerous possible cryptic species in the genus *Hyalella* [53], and a few in the presumed well-studied genus *Gammarus* [54], [55]). Equally, in the Isopoda, cryptic, reproductively-isolated species were indicated within the asellote *Acanthaspidia drygaskii* [56], in the sphaeromatidean *Serolis paradoxa* [57], and complexes of cryptic biological species were demonstrated in the asellote *Betamorpha fusiforis* [58], all using 16S rDNA analysis, the last in combination with 18S rRNA. It is to be expected that similar results would be found on closer study of the Tanaidacea.

**Figure 7:**
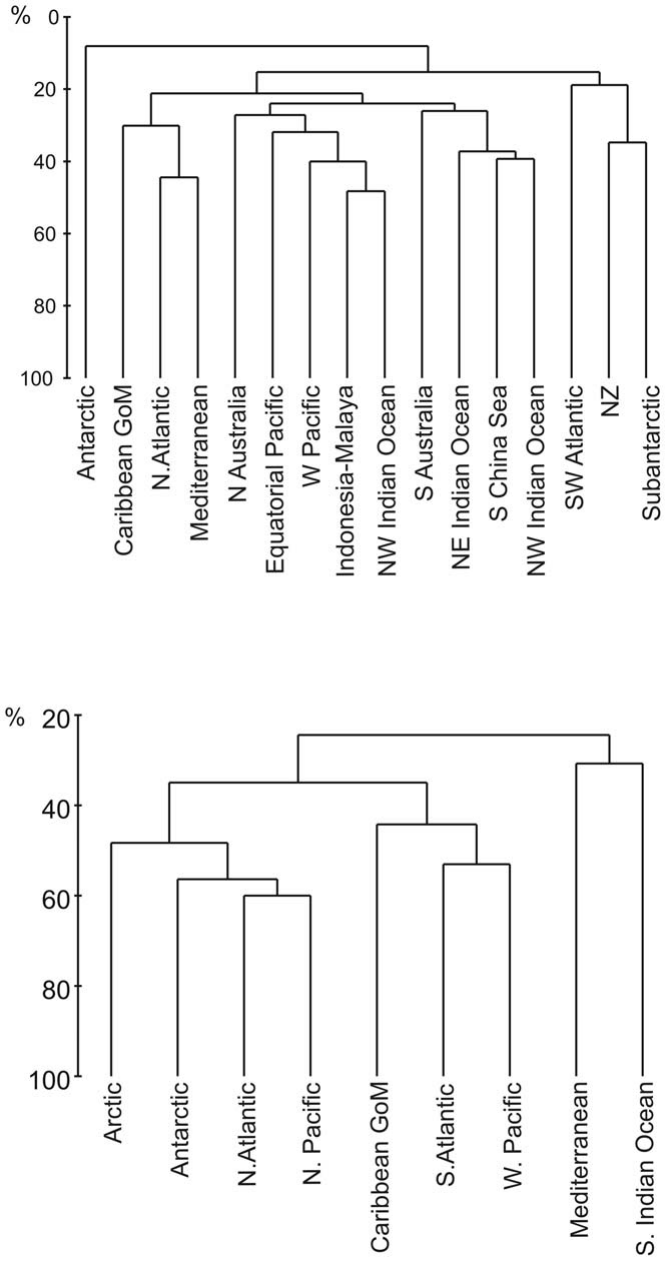
Cumulative curve of the number of species known (published) from Australian waters since the first published in 1882 (histogram bars illustrate the number published each year).

- that there are many regions of the World whose shallow-water tanaidacean faunas are as yet understudied (although material does exist in some museums). Study of the tanaidacean fauna of Australia over the last two decades, predominantly in shallow waters, has discovered an unprecedented and unrealized diversity, increasing the number of described species from 16 in 1996 to 117 by 2010 (Figure 7). Błażewicz-Paszkowycz and Bamber [38] found 57 of 65 species collected above 200 m in the Bass Strait, Australia, to be undescribed species. Comparable detailed study has yet to be undertaken for regions which might be presumed to show a related assemblage, such as southern Africa, India, and the Pacific Islands. In the case of the latter, the number of species known from New Caledonia has risen from zero to 13 in the past five years [59]; elsewhere, more recent studies [60], [61] have already increased the New Zealand shallow-water tanaidomorph fauna from two to 12 species.

Surprisingly, in what might be thought a better-studied area, recent investigations of the eastern Mediterranean fauna [29] found high densities of previously undescribed tanaidacean species [60], despite the history of study of that basin going back to the 19th century. Equally, a number of species has been added to the shallow-water British fauna over the last 25 years [46], [62], [63], despite the long history of study in this region, suggesting that understudied regions are not necessarily remote

- that the relative significance of morphological (or molecular) characters in phylogeny is presently not well understood. Criteria for distinguishing super-specific taxa are often almost arbitrary, as is a clear understanding over what defines (or distinguishes) a genus or a family. It is inevitably the case that certain features of morphology evolve more rapidly in response to environmental selection pressures (e.g. body size, presence of functional eyes, or mouthpart morphology) or to ecological (life-style) selection pressures (e.g. development of pleopods, body elongation, and again mouthpart morphology), while others are less so influenced, and give a better phylogenetic signal (e.g. number of oostegites, or fusion of the pereopod ischium).

Interpretation of the development of, or history of, biodiversity and its zoogeographical interpretation relies understanding the genetic relationships between species, and therefore between genera and even higher taxa. If researchers on tanaidacean taxonomy are unclear about the level of, and degree of change in, characters which distinguish taxa, then it becomes increasingly difficult to define and to associate those taxa, and thus to determine biodiversity.

As a result of all of these, the current World list of described species is likely to be at least an order-of-magnitude too low. Marked advances have been made in recent years, but progress in understanding the tanaidacean faunas, particularly from more remote regions, will continue to be restricted by the lack of competent tanaidacean researchers and taxonomists, the lack of appropriate sampling and sample treatment, and of course the lack of funding to pursue such pure science.

Nevertheless, it is evident from our current knowledge that the Tanaidacea form a very diverse order of the Peracarida, potentially rivalling in diversity the Amphipoda and Isopoda globally, and of considerable ecological significance in certain regions and habitats.
